# Supplementary material for: The microbial metabolite desaminotyrosine protects against graft-versus-host disease via mTORC1 and STING-dependent intestinal regeneration
Source: Nat Commun. 2025 Oct 20;16:9282. doi: 10.1038/s41467-025-65180-6 (PMC12537870; doi:10.1038/s41467-025-65180-6)
Supplement: Supplementary file 1 — Supplementary Information [file 41467_2025_65180_MOESM1_ESM.pdf]

Table S1

**Patients – no.**		50
**Mean age at HSCT – yrs ± SE**		56.5 ± 1.5
**Male/Female Sex – no. (%)**		27 (54)/23 (46)
**Diagnosis – no. (%)**	Acute leukaemia	22 (44)
	MDS/MPN	19 (38)
	NHL	5 (10)
	other	4 (8)
**Donor type – no. (%)**	Unrelated	35 (70)
	Sibling	5 (10)
	Haploidentical	10 (20)
**Conditioning – no. (%)**	Ablative	7 (14)
	Reduced intensity	43 (86)
**Stem cell source – no. (%)**	PBSC	42 (84)
	BM	8 (16)
**High DAT levels – no. (%)**		22 (44)
**High ICA levels – no. (%)**		10 (20)
**2 year relapse incidence (%)**		10 (20)
**2 year TRM incidence (%)**		10 (20)
**2 year survival (%)**		31 (62)

Patient characteristics. HSCT denotes hematopoietic stem cell transplantation, SE standard error, MDS myelodysplastic syndrome, MPN myeloproliferative neoplasia, NHL Non-Hodgkin lymphoma, PBSC peripheral-blood stem cell, BM bone marrow, and TRM transplantation-related mortality. Diseases categorized as “other” include aplastic anemia and myelosarcoma.

Table S2

Antigen	Fluorophore	RRID	Company	Dilution
m CD25	PE	AB_395101	BD	1:200
m CD4	Pacific Blue	AB_2739450	BD	1:200
m CD8a	PerCP/Cyanine5.5	AB_394081	BD	1:200
m Ly-6C	Pacific Blue	AB_1732090; AB_1732079	Biolegend	1:200
m CD11b	PE/Cyanine7	AB_394491	BD	1:200
m CD326	BUV395	AB_2740020	BD	1:200
m CD45	Alexa Fluor 700	AB_493714; AB_493715	Biolegend	1:250
m CD11b	BUV395	AB_2738276	BD	1:1000
m CD8a	BUV395	AB_2732919	BD	1:200
m CD3	FITC	AB_312660; AB_312661	Biolegend	1:200
m CD279 (PD-1)	PE	AB_1877232; AB_1877231	Biolegend	1:250
m CD4	PerCP/Cyanine5.5	AB_893330; AB_893324	Biolegend	1:200
m IFN-γ	PE/Cyanine7	AB_1595591; AB_2295770	Biolegend	1:750
m (C57BL) H-2Kb	Brilliant Violet 421	AB_2876430	Biolegend	1:200
m (BALB/c) H-2Kd	Brilliant Violet 421	AB_2565656	Biolegend	1:200
m/h FoxP3	Alexa Fluor 647	AB_439749; AB_439750	Biolegend	1:100
m I-A/I-E	FITC	AB_313320; AB_313321	Biolegend	1:1500
m CD11c	PE	AB_313776; AB_313777	Biolegend	1:200
m F4/80	PE/Cyanine7	AB_893490; AB_893478	Biolegend	1:800
m Ly-6C	Brilliant Violet 421	AB_2562177; AB_2562178	Biolegend	1:1000
m CD103	Brilliant Violet 605	AB_2629724	Biolegend	1:1000
m Ly-6G	Alexa Fluor 647	AB_1134162; AB_1134159	Biolegend	1:1500
m CD3	APC/Cyanine7	AB_2057374; AB_2242784	Biolegend	1:200
m CD86	PE	AB_313150; AB_313151	Biolegend	1:200
m CD80	PerCP/Cyanine5.5	AB_893406; AB_2291392	Biolegend	1:200
m CD11c	APC/Cyanine7	AB_830646; AB_830649	Biolegend	1:300
m CD45	PerCP/ Cyanine5.5	AB_893344; AB_893340	Biolegend	1:500
m CD3	APC	AB_2561455; AB_2561456	Biolegend	1:200
m CD3	Brilliant Violet 421	AB_10900227; AB_2562553	Biolegend	1:200
m CD117	PE	AB_2734235	Biolegend	1:200
h CD14	Brilliant Violet 421	AB_2810579	Biolegend	1:200
h CD209	APC	AB_1134055	Biolegend	1:200
h CD11c	PerCP	AB_2566656	Biolegend	1:200
h CD80	BV605	AB_11123909	Biolegend	1:200
h CD86	BV711	AB_2565834	Biolegend	1:200
h HLA-DR	FITC	Cat. 980402	Biolegend	1:200
h TNFa	BUV395	AB_2738533	BD	1:100
h CD25	BUV737	AB_2870132	BD	1:100
h CD8a	BUV805	AB_2871326	BD	1:100
h PD-1	Brilliant Violet 421	AB_2721517	Biolegend	1:100
h TIM-3	Brilliant Violet 605	AB_2741099	BD	1:100
h T-bet	Brilliant Violet 650	AB_2738616	BD	1:100
h CD4	Brilliant Violet 711	AB_2737965	BD	1:100
h CD3	Brilliant Violet 785	AB_11219196	Biolegend	1:100
h Perforin	FITC	AB_493252	Biolegend	1:100
h IFNy	BB700	AB_2744484	BD	1:100
h Granzyme B	PE-Dazzle 594	AB_2728382	Biolegend	1:100
Foxp3	PE-Cy5	AB_10597134	Thermo Fisher	1:50
TotalSeq™-B0301 anti-mouse Hashtag 1	Oligo Hashtag	AB_2814067	Biolegend	1:100
TotalSeq™-B0302 anti-mouse Hashtag 2	Oligo Hashtag	AB_2814068	Biolegend	1:100
TotalSeq™-B0303 anti-mouse Hashtag 3	Oligo Hashtag	AB_2814069	Biolegend	1:100
TotalSeq™-B0304 anti-mouse Hashtag 4	Oligo Hashtag	AB_2814070	Biolegend	1:100
TotalSeq™-B0305 anti-mouse Hashtag 5	Oligo Hashtag	AB_2814071	Biolegend	1:100
TotalSeq™-B0306 anti-mouse Hashtag 6	Oligo Hashtag	AB_2814072	Biolegend	1:100

Overview of all utilized antibodies for flow cytometry and the used dilutions.

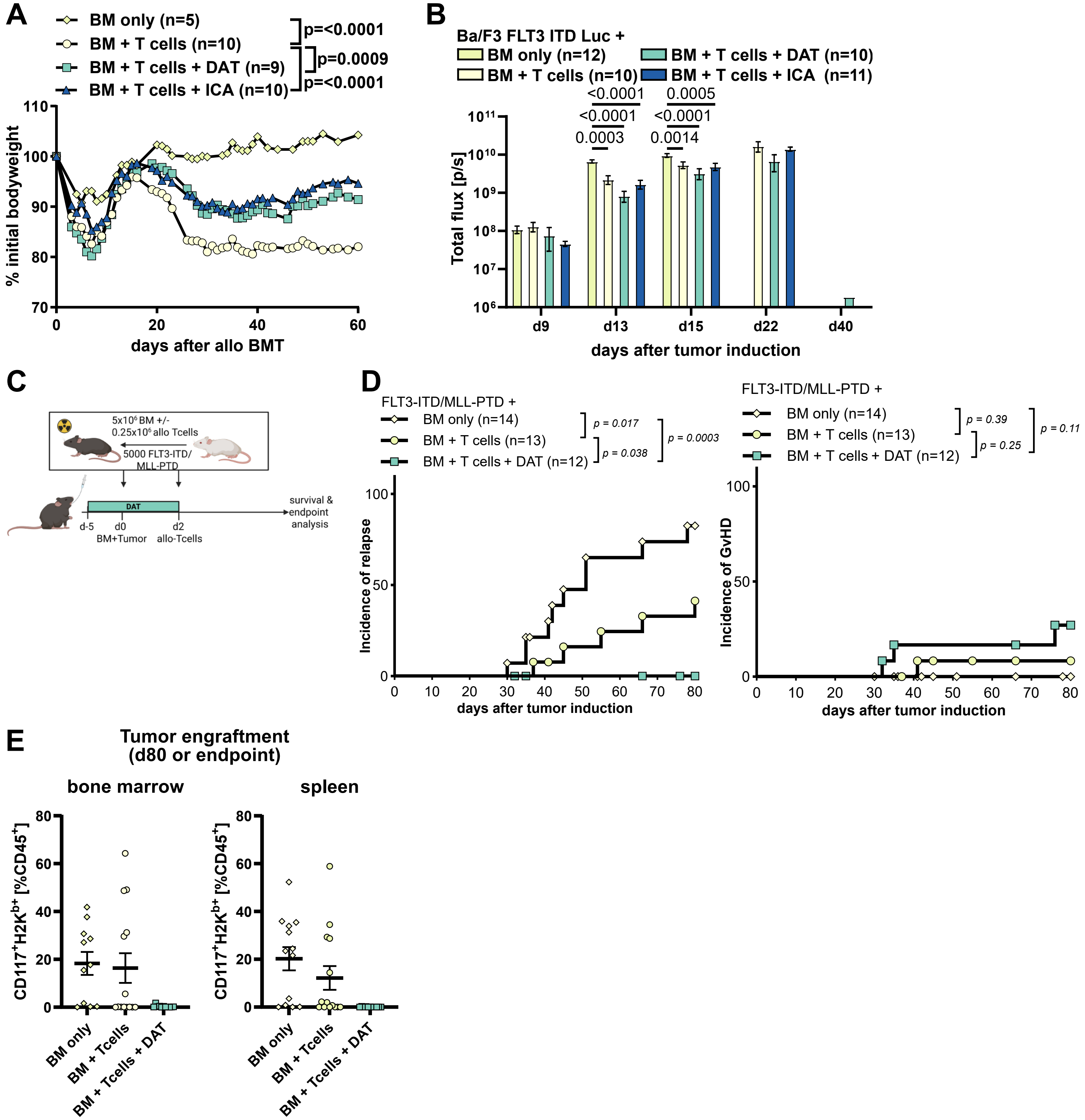
A
BM only (n=5)
BM + T cells (n=10)
BM + T cells + DAT (n=9)
BM + T cells + ICA (n=10)
p=<0.0001
p=0.0009
p=<0.0001
% initial bodyweight
days after allo BMT
B
Ba/F3 FLT3 ITD Luc +
BM only (n=12)
BM + T cells (n=10)
BM + T cells + DAT (n=10)
BM + T cells + ICA (n=11)
Total flux [p/s]
days after tumor induction
C
5x10^6 BM +/- 0.25x10^6 allo Tcells
5000 FLT3-ITD/MLL-PTD
DAT
survival & endpoint analysis
d-5
d0
BM+Tumor
d2
allo-Tcells
D
FLT3-ITD/MLL-PTD +
BM only (n=14)
BM + T cells (n=13)
BM + T cells + DAT (n=12)
p = 0.017
p = 0.038
p = 0.0003
Incidence of relapse
days after tumor induction
p = 0.39
p = 0.25
p = 0.11
Incidence of GvHD
E
Tumor engraftment (d80 or endpoint)
bone marrow
spleen
CD117+H2Kb+ [%CD45+]
BM only
BM + Tcells
BM + Tcells + DAT

Figure S1

Fig. S1: supporting weight loss and BLI data, related to Fig. 1. (A) Corresponding weight loss of mice shown in Fig. 1 E. Weight curves were analyzed by calculating the area under the curve (AUC) and ordinary one-way ANOVA with Dunnett's correction for multiple comparisons. **(B)** Leukemia cell expansion of mice shown in Fig. 1F-I was monitored by bioluminescence imaging. Quantification of BLI signal on depicted days from two independent experiments. P-values were calculated using two-way ANOVA with Dunnett's correction for multiple comparisons. **(C)** Experimental setting for the FLT3-ITD/MLL-PTD tumor model. **(D)** Incidence of relapse or GvHD of mice following tumor transplantation and allo-BMT. P-values were generated by Log-rank test. **(E)** The tumor burden in bone marrow or spleen was analysed in mice reaching human endpoint or at the end of the experiment. Data is shown as mean ± SEM. **(C)** Created in BioRender. Göttert, S. (2025) https://BioRender.com/wg9py37.

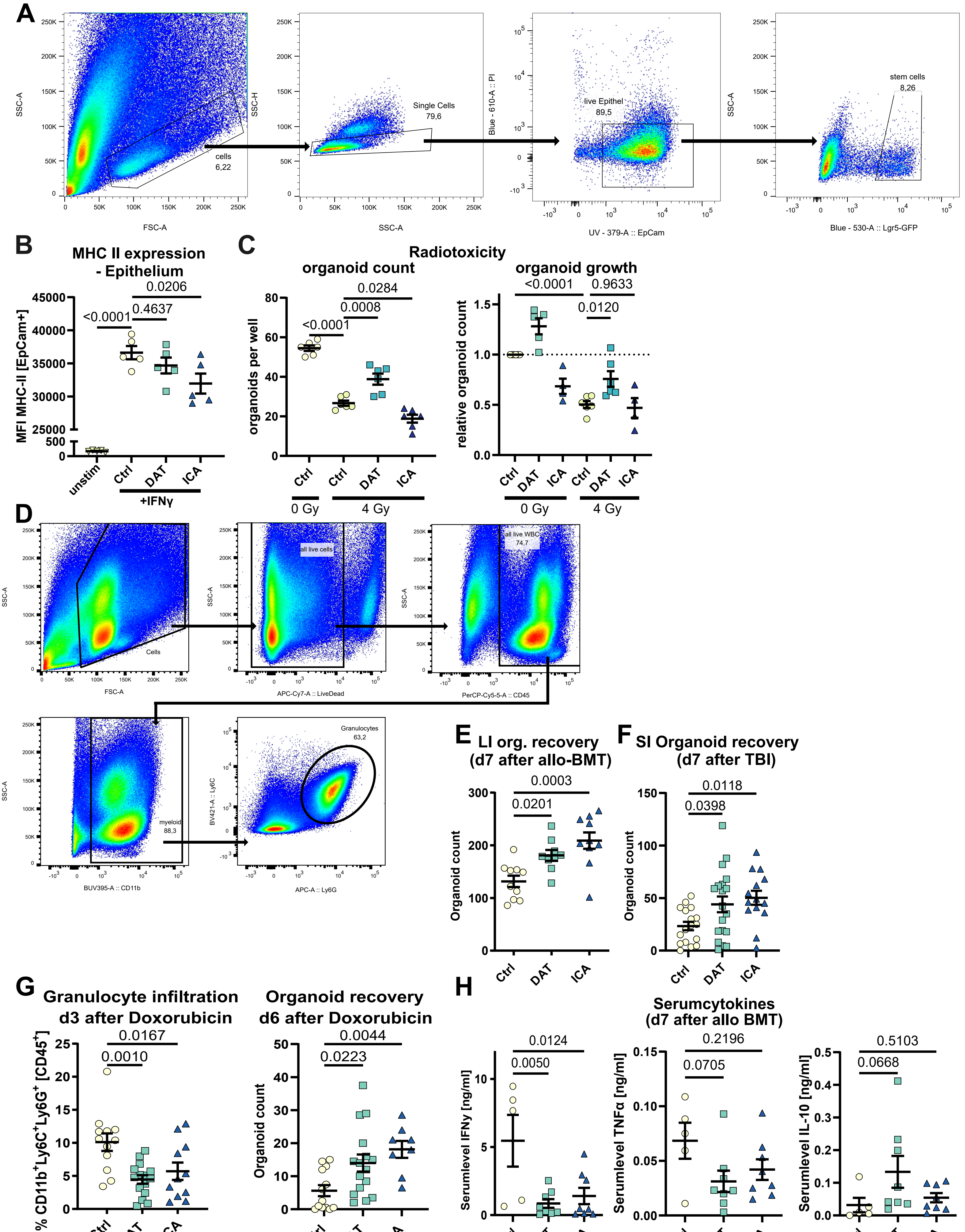
A
cells 6,22
Single Cells 79,6
live Epithel 89,5
stem cells 8,26
FSC-A
SSC-A
SSC-H
Blue - 610-A :: PI
UV - 379-A :: EpCam
Blue - 530-A :: Lgr5-GFP
B
MHC II expression - Epithelium
MFI MHC-II [EpCam+]
<0.0001
0.4637
0.0206
unstim
Ctrl
DAT
ICA
+IFNγ
C
Radiotoxicity
organoid count
organoids per well
<0.0001
0.0008
0.0284
0 Gy
4 Gy
organoid growth
relative organoid count
<0.0001
0.9633
0.0120
D
Cells
all live cells
all live WBC 74,7
myeloid 88,3
Granulocytes 63,2
APC-Cy7-A :: LiveDead
PerCP-Cy5-5-A :: CD45
BUV395-A :: CD11b
BV421-A :: Ly6C
APC-A :: Ly6G
E
LI org. recovery (d7 after allo-BMT)
Organoid count
0.0201
0.0003
F
SI Organoid recovery (d7 after TBI)
0.0398
0.0118
G
Granulocyte infiltration d3 after Doxorubicin
% CD11b+Ly6C+Ly6G+ [CD45+]
0.0010
0.0167
Organoid recovery d6 after Doxorubicin
0.0223
0.0044
H
Serumcytokines (d7 after allo BMT)
Serumlevel IFNy [ng/ml]
0.0050
0.0124
Serumlevel TNFα [ng/ml]
0.0705
0.2196
Serumlevel IL-10 [ng/ml]
0.0668
0.5103

Figure S2

Fig. S2: supporting *in vitro* organoid and *in vivo* data, related to Fig. 2. (A) Gating strategy used for FACS analyses of epithelial cells derived from murine small intestinal organoids. **(B)** Surface expression of MHC class II on EpCAM+ epithelial cells after IFNγ stimulation. Murine small intestinal organoids were seeded and stimulated with indicated metabolites or control for 3 days. Afterwards, indicated groups were stimulated with 2.5ng/ml IFNγ alone or in combination with metabolites. The expression of MHC II was determined after additional 3 days of IFNγ stimulation. Pooled data from 5 experiments. **(C)** Left: Number of viable organoids alone or after irradiation with 4 Gy with additional metabolite or control treatment assessed on day 6 of culture. N=6 wells from 2 independent experiments. P-values were calculated with ordinary one-way ANOVA with Dunnett's correction for multiple comparisons. Right: Organoid numbers were assessed after passage. N=4 (0Gy ICA, 4 Gy ICA), 5 (0Gy DAT) or 6 (0Gy Ctr, 4Gy Ctrl, 4Gy DAT) experiments. P-values were calculated by ordinary one-way ANOVA with Dunnett's correction for multiple comparisons. **(D)** Gating strategy utilized to determine the infiltration of neutrophil granulocytes into the lamina propria of the ileum after damage induction. **(E)** Count of regenerated large intestinal organoids from 300 seeded crypts per drop. Pooled data from 3 independent experiments (n=9 (DAT) or 10 (Ctrl, ICA)). P-values were calculated by ordinary one-way ANOVA with Dunnett's correction for multiple comparisons. **(F)** Number of viable organoids generated from 200 crypts isolated on day 7 after TBI. Pooled data from 4 (n=14 (ICA), 17 (Ctrl) or 19(DAT)) independent experiments. P-values were calculated by ordinary one-way ANOVA with Dunnett's correction for multiple comparisons. **(G)** Granulocyte infiltration (left) and organoid recovery (right) after intestinal mucositis was induced by intraperitoneal injection of the chemotherapeutic doxorubicin. C57BL/6 mice were treated with metabolites from day 7 before damage induction and intraperitoneal injections of doxorubicin were performed on day 0. Barrier damage was assessed on day 3 by granulocyte infiltration (n=12 (Ctrl), 15 (DAT), 11 (ICA)) and organoid recovery was assessed on day 6 after doxorubicin injection (n=13 (Ctrl), 16 (DAT), 8 (ICA)). P-values were calculated by ordinary one-way ANOVA with Dunnett's correction for multiple comparisons. **(H)** Serum cytokine concentration determined in C57BL/6 mice treated as in Fig. 2 G by cytokine bead array. N=5 (Ctrl) or 8 (DAT and ICA) P-values were calculated by ordinary one-way ANOVA with Dunnett's correction for multiple comparisons.

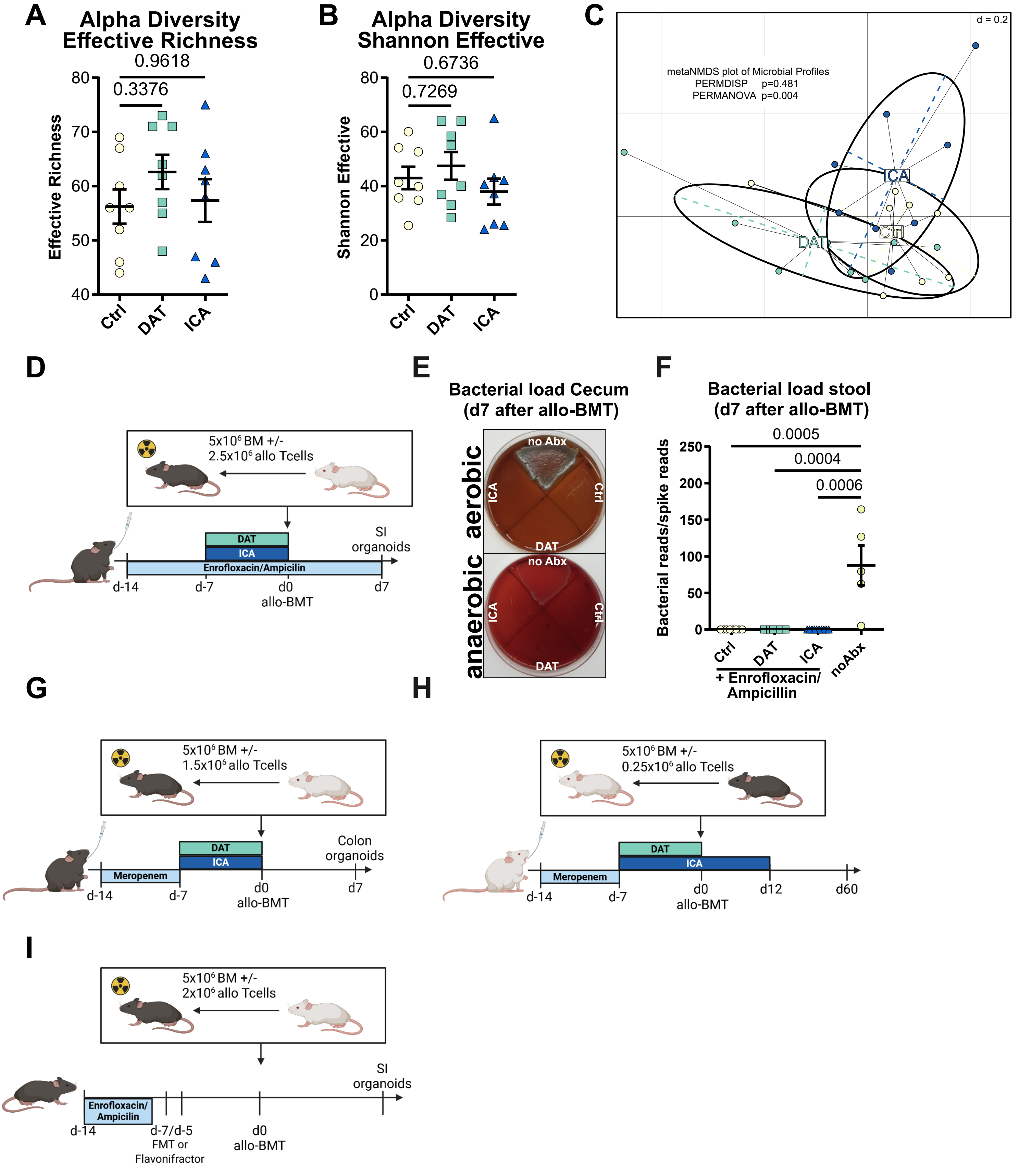
A
Alpha Diversity
Effective Richness
0.9618
0.3376
Effective Richness
80
70
60
50
40
Ctrl
DAT
ICA
B
Alpha Diversity
Shannon Effective
0.6736
0.7269
Shannon Effective
80
60
40
20
0
Ctrl
DAT
ICA
C
d = 0.2
metaNMDS plot of Microbial Profiles
PERMDISP p=0.481
PERMANOVA p=0.004
ICA
Ctrl
DAT
D
5x10⁶ BM +/-
2.5x10⁶ allo Tcells
DAT
ICA
Enrofloxacin/Ampicilin
SI
organoids
d-14
d-7
d0
allo-BMT
d7
E
Bacterial load Cecum
(d7 after allo-BMT)
aerobic
anaerobic
no Abx
ICA
Ctrl
DAT
F
Bacterial load stool
(d7 after allo-BMT)
Bacterial reads/spike reads
250
200
150
100
50
0
0.0005
0.0004
0.0006
Ctrl
DAT
ICA
noAbx
+ Enrofloxacin/
Ampicillin
G
5x10⁶ BM +/-
1.5x10⁶ allo Tcells
DAT
ICA
Meropenem
Colon
organoids
d-14
d-7
d0
allo-BMT
d7
H
5x10⁶ BM +/-
0.25x10⁶ allo Tcells
DAT
ICA
Meropenem
d-14
d-7
d0
allo-BMT
d12
d60
I
5x10⁶ BM +/-
2x10⁶ allo Tcells
Enrofloxacin/
Ampicilin
SI
organoids
d-14
d-7/d-5
FMT or
Flavonifractor
d0
allo-BMT

Figure S3

Fig. S3: supporting microbiome data, related to Fig. 3: Mice were treated with metabolites as described in Fig. 2 G for 7 days. Stool samples of metabolite and carrier treated mice (n=8) were collected and analyzed by 16SrRNA sequencing. Alpha diversity indicated as effective richness **(A)** and Shannon effective **(B)** are shown. P-values were calculated using ordinary one-way ANOVA with Dunnett's correction for multiple comparisons. **(C)** Non-metric multidimensional scaling (NMDS) biplot showing distance between indicated groups. **(D)** Experimental design for metabolite treatment, allo-BMT and gut decontamination by ampicillin and enrofloxacin. **(E)** Gut decontamination after treatment with ampicillin/enrofloxacin was confirmed. Cecum content of mice treated in Fig. 3D was collected at the end of the experiment, resuspended in PBS and plated on blood agar plates cultivated under aerobic and anaerobic conditions for 48h or **(F)** 16S-rRNA of faecal samples from Abx-treated +/- metabolites and non-Abx-treated mice were analyzed and the bacterial load of each sample was graphed as the ratio of bacterial reads to spike reads (n=8 (Ctrl, DAT), 10 (ICA) or 5 (no ABX)). P-values were calculated by P-values were calculated by ordinary one-way ANOVA with Dunnett's correction for multiple comparisons. **(G)** Experimental design for metabolite treatment and allo-BMT following induction of dysbiosis by meropenem treatment. (H) Experimental design for metabolite treatment and allo-BMT following induction of dysbiosis by meropenem treatment. **(I)** Experimental design for gut decontamination by ampicillin and enrofloxacin followed by FMT or transplantation of flavonifractor plautii isolates before allo-BMT. **(D)** Created in BioRender. Göttert, S. (2025) https://BioRender.com/vmdiadk. **(G)** Created in BioRender. Göttert, S. (2025) https://BioRender.com/k16saeg. **(H)** Created in BioRender. Göttert, S. (2025) https://BioRender.com/4k3iffl. **(I)** Created in BioRender. Göttert, S. (2025) https://BioRender.com/pd4i5f3.

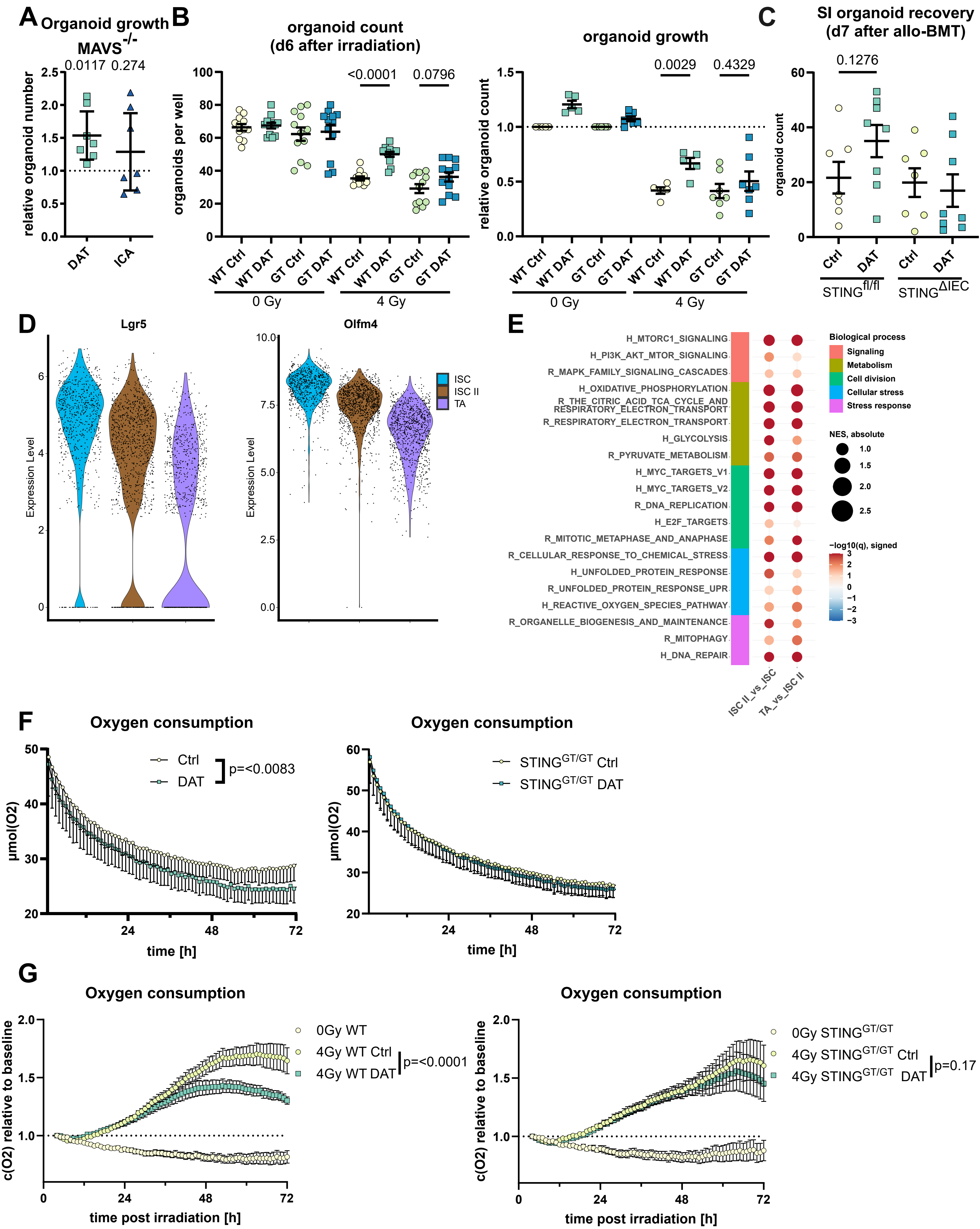

A
Organoid growth MAVS-/-
0.0117 0.274
relative organoid number
DAT
ICA
B
organoid count (d6 after irradiation)
<0.0001 0.0796
organoids per well
WT Ctrl
WT DAT
GT Ctrl
GT DAT
0 Gy
4 Gy
organoid growth
0.0029 0.4329
relative organoid count
C
SI organoid recovery (d7 after allo-BMT)
0.1276
organoid count
Ctrl
DAT
STING fl/fl
STING ΔIEC
D
Lgr5
Olfm4
Expression Level
ISC
ISC II
TA
E
H_MTORC1_SIGNALING
H_PI3K_AKT_MTOR_SIGNALING
R_MAPK_FAMILY_SIGNALING_CASCADES
H_OXIDATIVE_PHOSPHORYLATION
R_THE_CITRIC_ACID_TCA_CYCLE_AND RESPIRATORY_ELECTRON_TRANSPORT
R_RESPIRATORY_ELECTRON_TRANSPORT
H_GLYCOLYSIS
R_PYRUVATE_METABOLISM
H_MYC_TARGETS_V1
H_MYC_TARGETS_V2
R_DNA_REPLICATION
H_E2F_TARGETS
R_MITOTIC_METAPHASE_AND_ANAPHASE
R_CELLULAR_RESPONSE_TO_CHEMICAL_STRESS
H_UNFOLDED_PROTEIN_RESPONSE
R_UNFOLDED_PROTEIN_RESPONSE_UPR
H_REACTIVE_OXYGEN_SPECIES_PATHWAY
R_ORGANELLE_BIOGENESIS_AND_MAINTENANCE
R_MITOPHAGY
H_DNA_REPAIR
ISC II_vs_ISC
TA_vs_ISC II
Biological process
Signaling
Metabolism
Cell division
Cellular stress
Stress response
NES, absolute
−log10(q), signed
F
Oxygen consumption
Ctrl
DAT
p=<0.0083
µmol(O2)
time [h]
STING GT/GT Ctrl
STING GT/GT DAT
G
Oxygen consumption
0Gy WT
4Gy WT Ctrl
4Gy WT DAT
p=<0.0001
c(O2) relative to baseline
time post irradiation [h]
0Gy STING GT/GT
4Gy STING GT/GT Ctrl
4Gy STING GT/GT DAT
p=0.17

Figure S4

Fig. S4: supporting in vitro organoid and sequencing data, related to Fig. 4. (A) The growth of SI organoids derived from MAVS$^{-/-}$ mice was assessed as in Fig. 2D. Pooled data from 7 independent experiments. P-values were calculated by one sample t-test. **(B)** Wildtype and STING-deficient organoids were treated with metabolites, irradiated with 4 Gy and analysed regarding organoid death (n=12 wells from 4 independent experiments) and growth (n=5 (WT) or 7 (STING$^{GT/GT}$) independent experiments) as described in Fig. S2C. P-values were calculated by unpaired t-test. **(C)** STING$^{fl/fl}$ or STING$^{\Delta IEC}$ mice underwent allo BMT and organoid regeneration was assessed on day 7 as described in Fig. 4D. Pooled data from 2 independent experiments. N=7 (Ctrl) or 8 (DAT) mice per group. **(D)** Expression level (natural logarithm of library-size-normalized counts) of Lgr5 or Olfm4 in indicated cell types. **(E)** Dotmap of GSEA results of selected pathways/gene sets for comparisons between cell types (cell types as described in Fig. 4F+G). Dots are colored by the negative $\log_{10}$ of the GSEA q-value (FDR), the sign indicates the direction of the regulation (up positive, down negative). The size of the dots corresponds to the normalized enrichment score (NES), a measure of gene set enrichment. Gene sets/pathways are derived from the Hallmark (H) and Reactome (R) gene set collections of MSigDB. **(F+G)** Media oxygen concentrations of metabolite or control-stimulated organoids alone or following irradiation with 4 Gy. Pooled data from 3 independent experiments (n=7 wells). P-values were calculated by unpaired t-test between AUC.

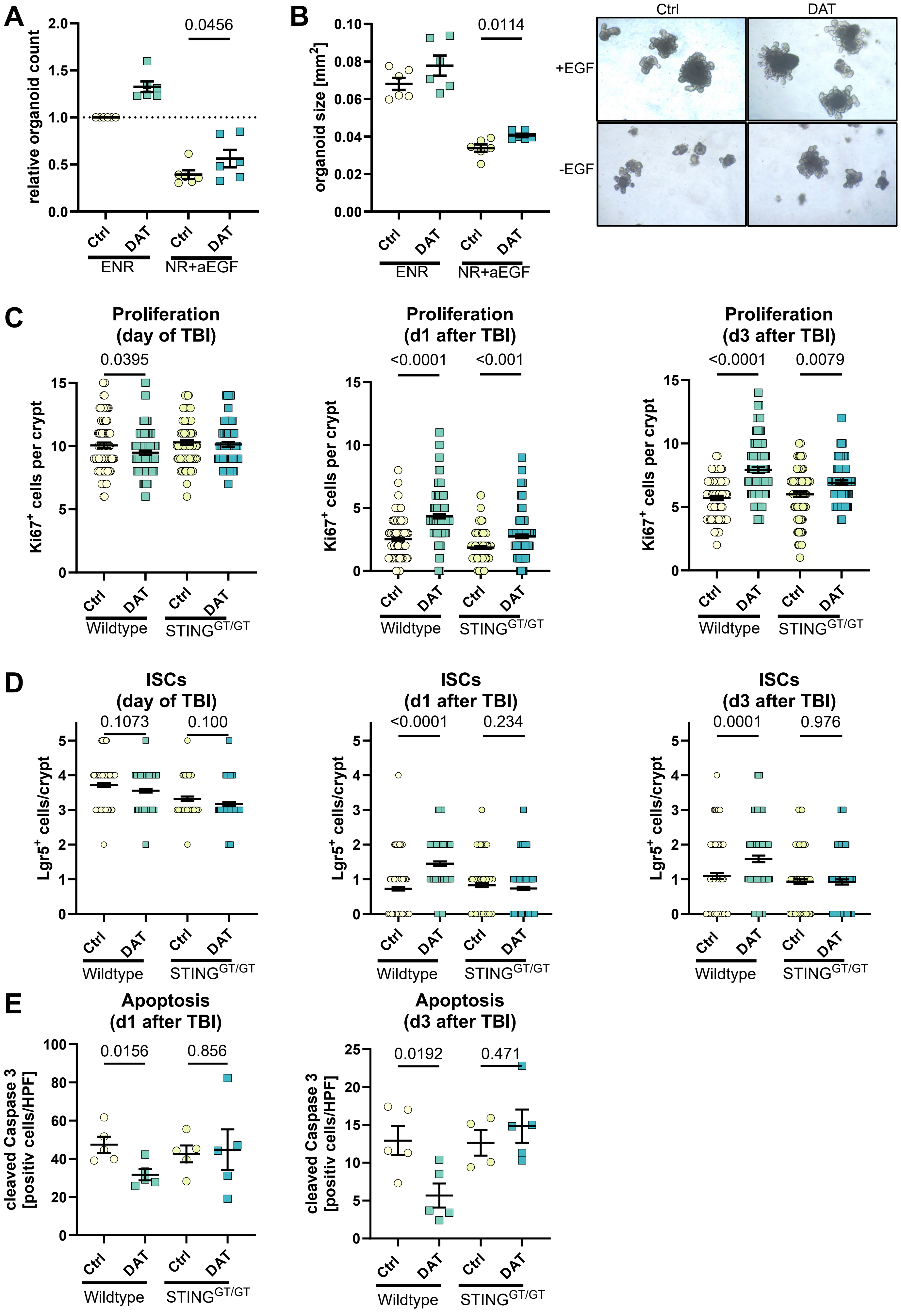
A
relative organoid count
0.0456
Ctrl
DAT
ENR
NR+aEGF
B
organoid size [mm2]
0.0114
Ctrl
DAT
+EGF
-EGF
ENR
NR+aEGF
C
Proliferation (day of TBI)
Proliferation (d1 after TBI)
Proliferation (d3 after TBI)
0.0395
<0.0001
<0.001
<0.0001
0.0079
Ki67+ cells per crypt
Wildtype
STINGGT/GT
D
ISCs (day of TBI)
ISCs (d1 after TBI)
ISCs (d3 after TBI)
0.1073
0.100
<0.0001
0.234
0.0001
0.976
Lgr5+ cells/crypt
E
Apoptosis (d1 after TBI)
Apoptosis (d3 after TBI)
0.0156
0.856
0.0192
0.471
cleaved Caspase 3 [positiv cells/HPF]

Figure S5

Fig. S5: supporting *in vitro* organoid data and *in vivo* data, related to Fig. 5. Murine SI organoid growth was assessed as described in Fig. 5F in ENR or NR media without EGF. Additionally, endogenous EGF produced by the organoids was blocked by addition of 0.5µg/ml EGF blocking antibody. **(A)** Organoid growth was assessed 3 days after passaging. N=6 independent experiments. **(B)** Size determination of organoids after 6 days of indicated treatments. N=6 independent experiments. **(C)** Number of Ki67-positive proliferating cells per large intestinal crypt following metabolite treatment alone or following TBI at the indicated time points. Pooled data from 2 independent experiments (d0: n=90 (WT Ctrl), 88 (WT DAT), 127 (GT Ctrl), 70 (GT DAT); d1: n=176 (WT Ctrl), 131 (WT DAT), 150 (GT Ctrl), 151 (GT DAT); d3: n=84 (WT Ctrl), 94 (WT DAT), 93 (GT Ctrl), 73 (GT DAT)). **(D)** Number of Lgr5-positive ISCs per large intestinal crypt following metabolite treatment alone or following TBI at the indicated time points. Pooled data from 2 independent experiments (d0: n=139 (WT Ctrl), 101 (WT DAT), 63 (GT Ctrl), 145 (GT DAT); d1: n=239 (WT Ctrl), 155 (WT DAT), 208 (GT Ctrl), 232 (GT DAT); d3: n=161 (WT Ctrl), 117 (WT DAT), 195 (GT Ctrl), 142 (GT DAT)). **(E)** Number of cleaved Caspase-3-positive cells per HPF in large intestine following metabolite treatment alone and TBI at the indicated time points. Pooled data from 2 independent experiments (n=4 (d3 $STING^{GT/GT}$ Ctrl) or 5). P-values were calculated by unpaired t-test.

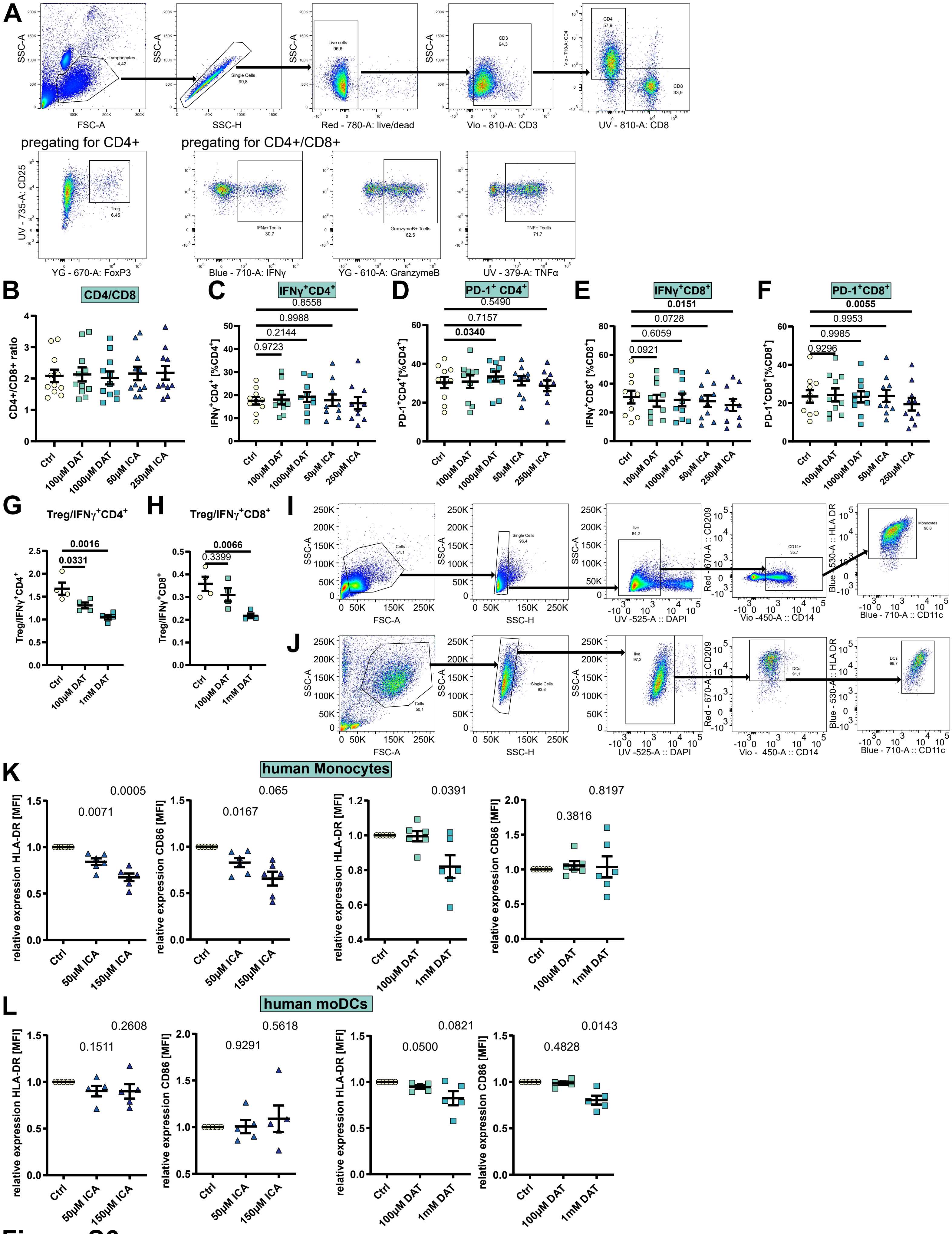
A
pregating for CD4+
pregating for CD4+/CD8+
B
CD4/CD8
C
IFNγ+CD4+
D
PD-1+ CD4+
E
IFNγ+CD8+
F
PD-1+CD8+
G
Treg/IFNγ+CD4+
H
Treg/IFNγ+CD8+
I
J
K
human Monocytes
L
human moDCs

Figure S6

Fig. S6: supporting flow cytometry data of human immune cells, related to Fig. 7. (A) Gating strategy used for *in vitro* stimulated human T cells utilized in Fig. 7 A-H. **(B)** Ratio of CD4+ to CD8+ T cells after stimulation with depicted metabolites as described in Fig. 7. **(C-F)** Percentage of PD-1^{+} or IFNγ^{+} $CD4^{+}$ or $CD8^{+}$ T cells. Pooled data from 10 healthy individuals. P values calculated by using ordinary one-way ANOVA for paired data with Dunnett's correction for multiple comparisons. **(G+H)** Murine panT cells were isolated from spleenocytes of C57Bl/6 mice and stimulated with anti-CD3/CD28 beads and 30U/ml murine IL-2 alone or in combination with DAT. Ratios of CD4+$CD25^{high}$ Tregs to IFNγ+ CD4+ **(G)** or IFNγ+ CD8+ **(H)** T cells. **(I-L)** Human antigen presenting cells were stimulated with metabolites and the surface expression of antigen presenting and co-stimulatory molecules determined. CD14+ monocytes were isolated from PBMCs of healthy volunteers and stimulated for 48h with indicated metabolites and analyzed by FACS. Alternatively, monocytes were differentiated into monocyte-derived DCs for 7 days, stimulated with indicated metabolites and analyzed by FACS. Gating strategies to define monocytes **(I)** or moDCs **(J)**. Surface expression of CD86 or HLA-DR on monocytes (n=6) **(K)** or moDCs (n=5) **(L)** after stimulation with indicated metabolites normalized to the surface expression of control. P-values were calculated by one-sample t-test.

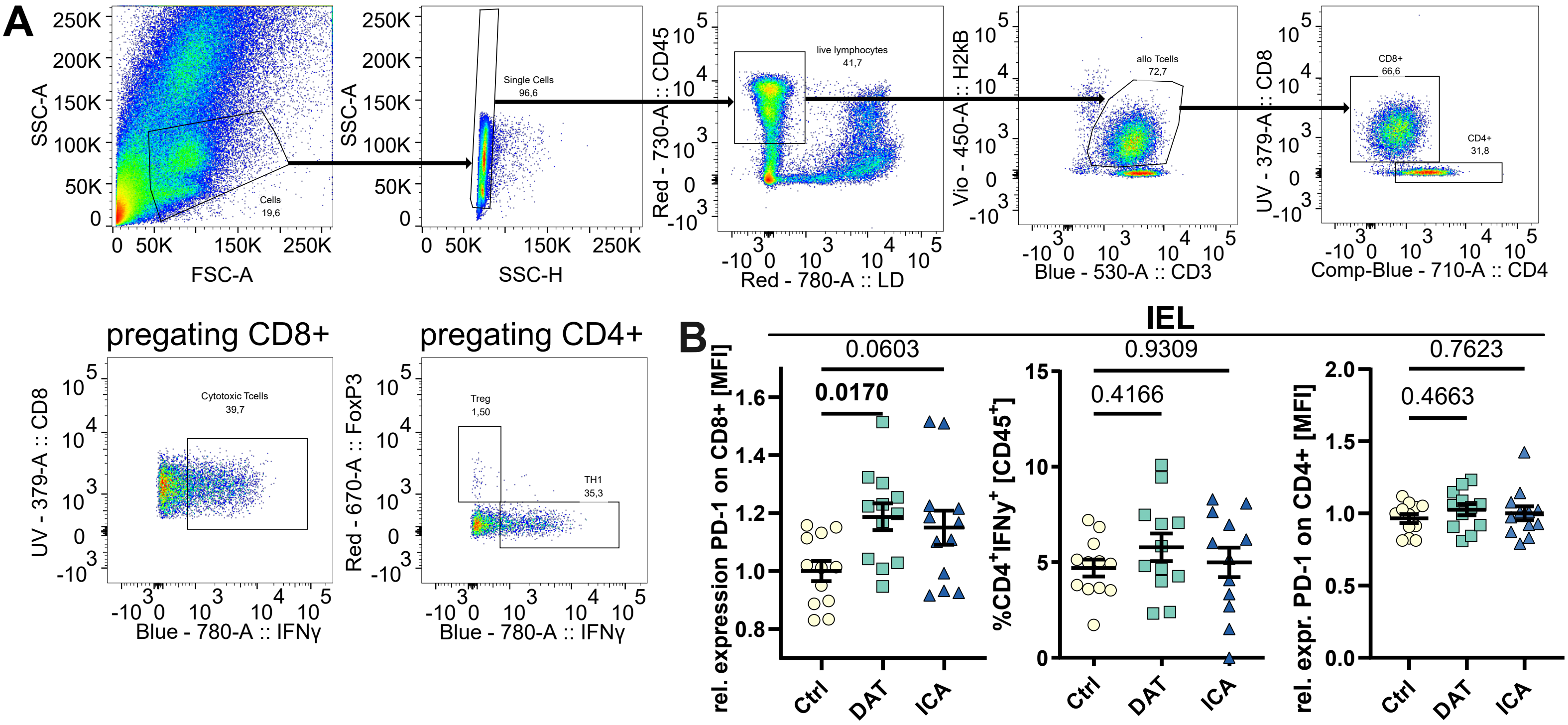
A
Cells 19,6
FSC-A
SSC-A
Single Cells 96,6
SSC-H
live lymphocytes 41,7
Red - 780-A :: LD
Red - 730-A :: CD45
allo Tcells 72,7
Blue - 530-A :: CD3
Vio - 450-A :: H2kB
CD8+ 66,6
CD4+ 31,8
Comp-Blue - 710-A :: CD4
UV - 379-A :: CD8
pregating CD8+
Cytotoxic Tcells 39,7
Blue - 780-A :: IFNγ
pregating CD4+
Treg 1,50
TH1 35,3
Red - 670-A :: FoxP3
B
IEL
0.0603
0.0170
rel. expression PD-1 on CD8+ [MFI]
0.9309
0.4166
%CD4+IFNy+ [CD45+]
0.7623
0.4663
rel. expr. PD-1 on CD4+ [MFI]
Ctrl
DAT
ICA

Figure S7

Fig. S7: supporting flow cytometry data of murine T cells, related to Fig. 7. (A) Gating strategy used for *in vivo* donor T cell analyses described in Fig. 7. BALB/c mice were orally treated with the metabolites and underwent allo-BMT as depicted in Fig. 1D. On day 7 mice were sacrificed and H-2K^{b+} donor T cells were analyzed by flow cytometry. **(B)** Additional results obtained from analyses of IEL (n=12 mice). P-values were calculated by ordinary one-way ANOVA with Dunnett's correction for multiple comparisons.

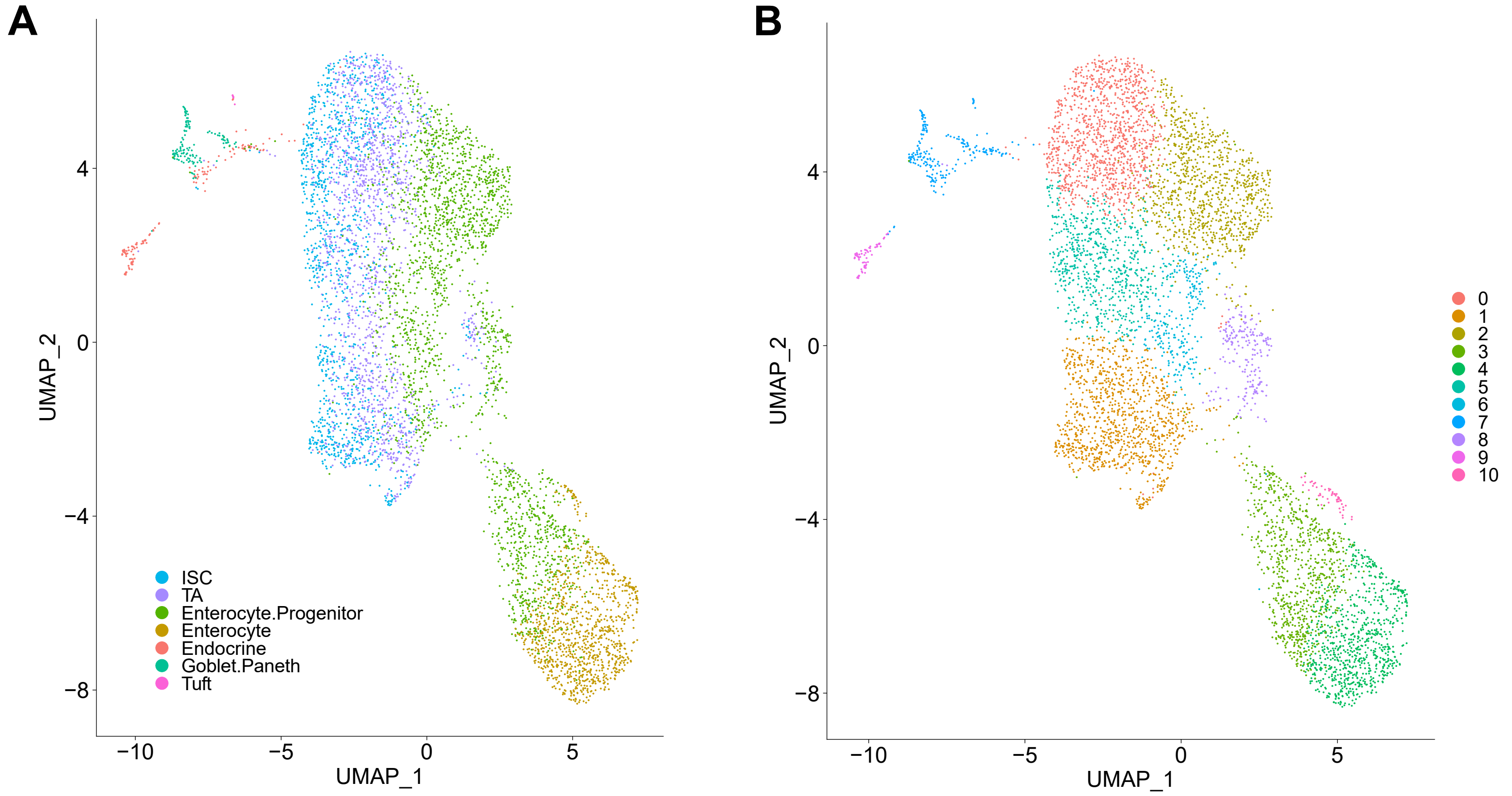

Figure S8, related to methods

Fig. S8: supporting data related to methods. (A) UMAP plot of SingleR cell type annotation. Plot of single cells in UMAP space, colored by SingleR cell type annotation. **(B)** UMAP plot of clustering results. Plot of single cells in UMAP space, colored by the results of graph-based clustering.

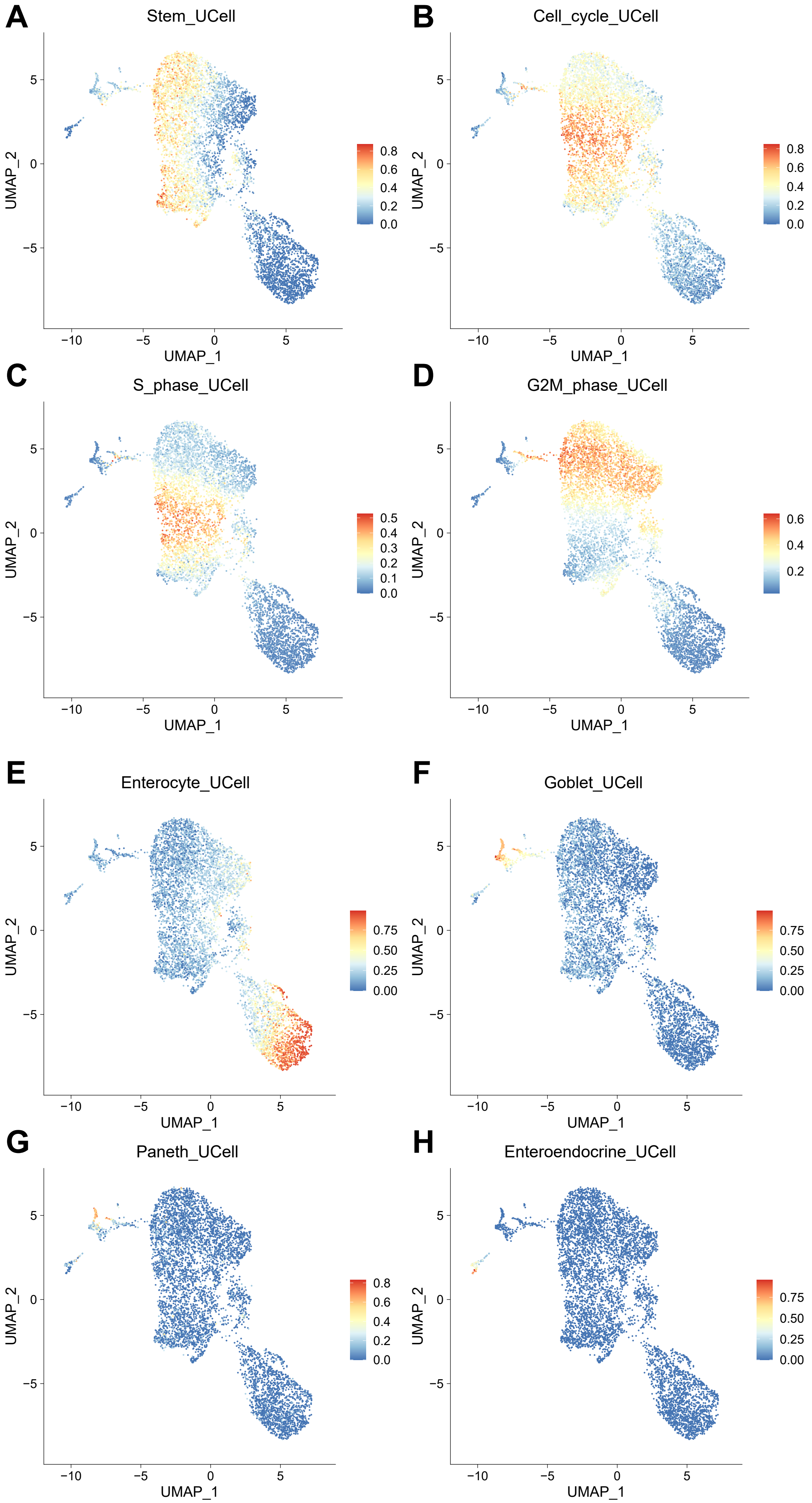

Figure S9, related to methods

Fig. S9: supporting data related to methods. (A-D) UMAP plots of UCell scores. Plots of single cells in UMAP space, colored by the UCell scores of the respective marker gene signature. Stem cells and cell cycle. **(E-H)** UMAP plots of UCell scores. Plots of single cells in UMAP space, colored by the UCell scores of the respective marker gene signature. Differentiated intestinal cell types.

A

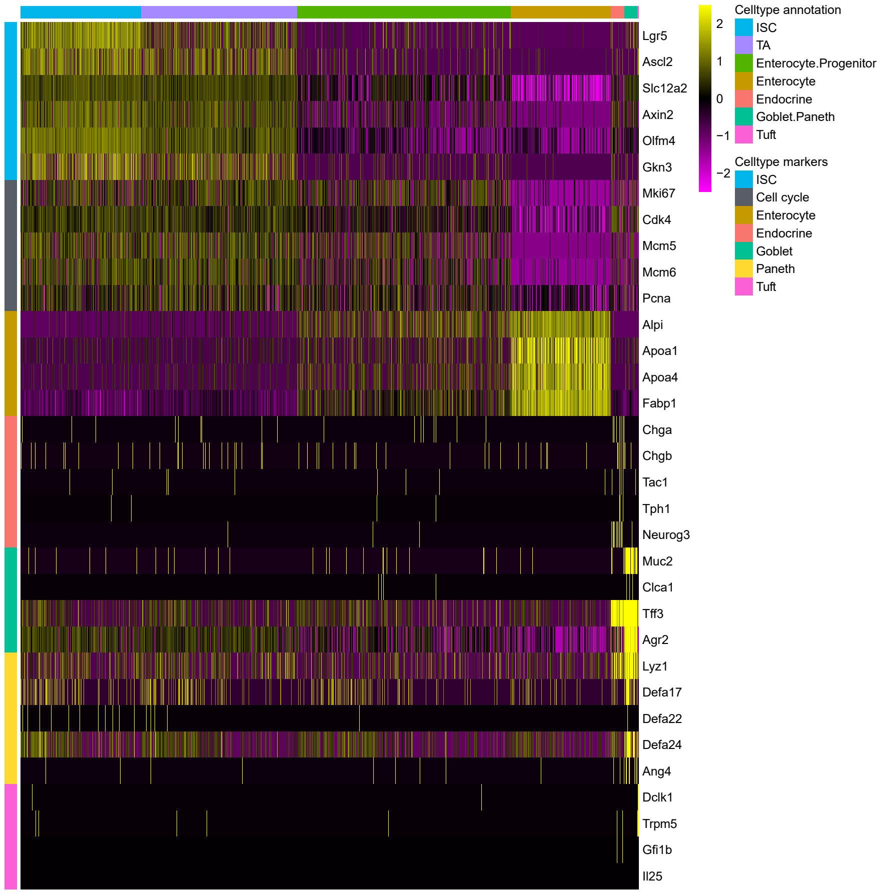

B

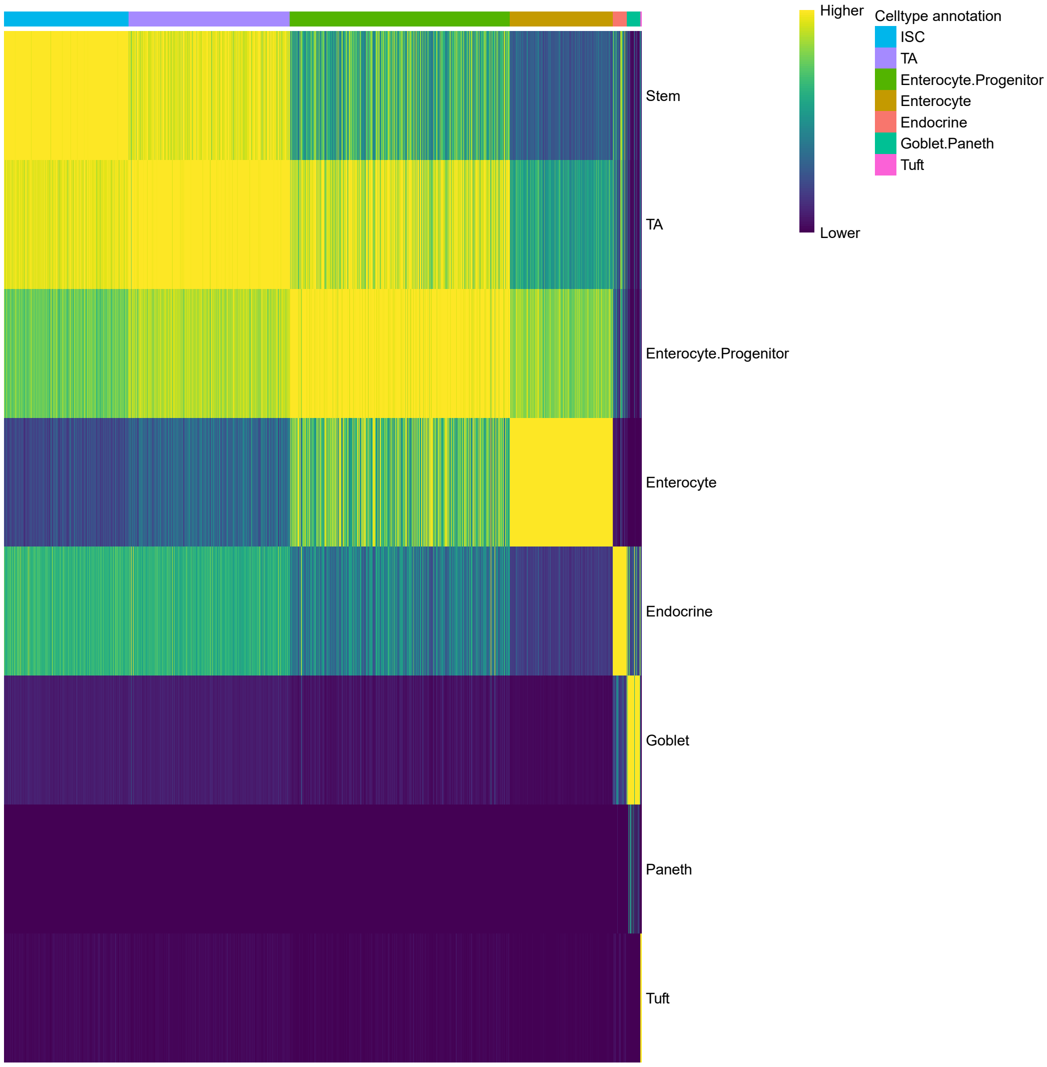

Figure S10, related to methods

Fig. S10: supporting data related to methods. (A) Heatmap of marker gene expression. Columns correspond to single cells, the bar on top provides the cell label obtained from SingleR annotation. Rows correspond to the gene expression of marker genes, where the bar to the left groups the marker genes according to their respective cell type/process. Gene expression is shown mean-centered, scaled to unit variance and clipped at values -2.5 and 2.5. **(B)** Heatmap of SingleR scores. Columns correspond to single cells, the bar on top provides the cell label obtained from SingleR annotation. Rows correspond to the SingleR scores for a given cell type label from the reference. Higher scores mean a higher correlation with and thus similarity to the reference label. Scores of single cells have been min-max normalized to lie within a [0, 1] interval, and transformed to the power of 3 to improve visibility of the dynamic range near 1 (see documentation of the SingleR package).

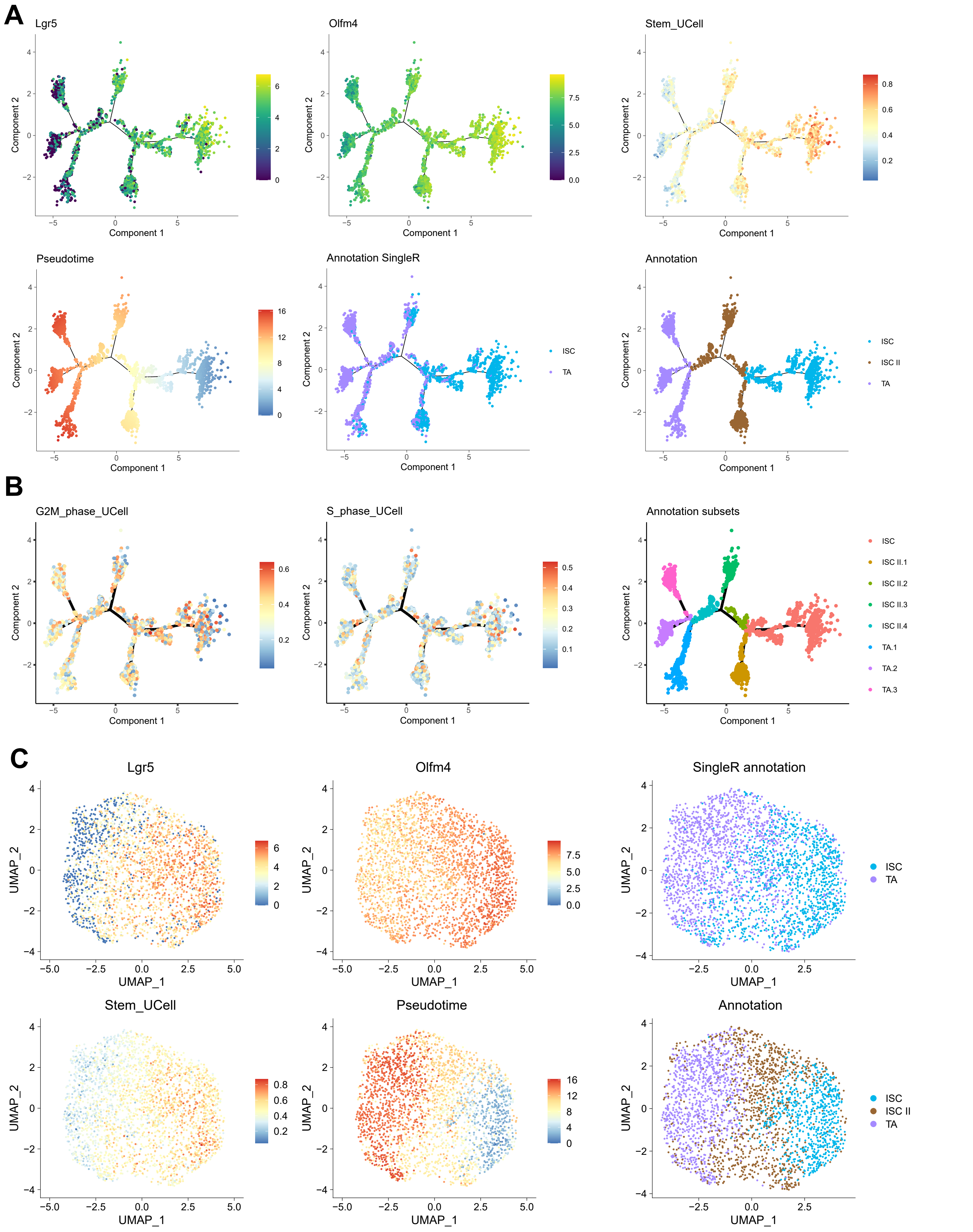

Figure S11, related to methods

Fig. S11: supporting data related to methods. (A) Plots of Monocle-based trajectory of ISC/TA subset. Stem-cell related features as well as trajectory pseudotime, SingleR annotation and final trajectory-based annotation are shown. Stem-cell marker expression is shown log-normalized, as described in the corresponding methods section. **(B)** Plots of Monocle-based trajectory of ISC/TA subset, ISC II and TA populations along trajectory Component 2. Panels show cell cycle related UCell scores as well as cell population subsets according to trajectory states when also considering branching in Component 2 for annotation. **(C)** UMAP plots of ISC/TA cell subset. Stem-cell related features as well as trajectory pseudotime, SingleR annotation and final trajectory-based annotation are shown. Stem-cell marker expression is shown log-normalized, as described in the corresponding methods section.
