# Supplementary material for: The microbial metabolite desaminotyrosine protects against graft-versus-host disease via mTORC1 and STING-dependent intestinal regeneration
Source: Nat Commun. 2025 Oct 20;16:9282. doi: 10.1038/s41467-025-65180-6 (PMC12537870; doi:10.1038/s41467-025-65180-6)
Supplement: Supplementary file 2 — Reporting Summary [file 41467_2025_65180_MOESM2_ESM.pdf]

nature portfolio

Corresponding author(s): Erik Thiele-Orberg
Hendrik Poeck

Last updated by author(s): Oct 7, 2025

Reporting Summary

Nature Portfolio wishes to improve the reproducibility of the work that we publish. This form provides structure for consistency and transparency in reporting. For further information on Nature Portfolio policies, see our Editorial Policies and the Editorial Policy Checklist.

Statistics

For all statistical analyses, confirm that the following items are present in the figure legend, table legend, main text, or Methods section.

n/a	Confirmed	
☐	☒	The exact sample size (*n*) for each experimental group/condition, given as a discrete number and unit of measurement
☐	☒	A statement on whether measurements were taken from distinct samples or whether the same sample was measured repeatedly
☐	☒	The statistical test(s) used AND whether they are one- or two-sided<br>*Only common tests should be described solely by name; describe more complex techniques in the Methods section.*
☐	☒	A description of all covariates tested
☐	☒	A description of any assumptions or corrections, such as tests of normality and adjustment for multiple comparisons
☐	☒	A full description of the statistical parameters including central tendency (e.g. means) or other basic estimates (e.g. regression coefficient) AND variation (e.g. standard deviation) or associated estimates of uncertainty (e.g. confidence intervals)
☐	☒	For null hypothesis testing, the test statistic (e.g. *F*, *t*, *r*) with confidence intervals, effect sizes, degrees of freedom and *P* value noted<br>*Give P values as exact values whenever suitable.*
☒	☐	For Bayesian analysis, information on the choice of priors and Markov chain Monte Carlo settings
☐	☒	For hierarchical and complex designs, identification of the appropriate level for tests and full reporting of outcomes
☒	☐	Estimates of effect sizes (e.g. Cohen's *d*, Pearson's *r*), indicating how they were calculated

Our web collection on statistics for biologists contains articles on many of the points above.

Software and code

Policy information about availability of computer code

Data collection	Data were collected using Microsoft Excel (16.0)
Data analysis	Graphpad Prism 10 (10.4.0), ggplot2 (version 3.3.5), patchwork (version 1.1.1), dplyr (version 1.0.7) stringr (version 1.4.0) and tidyr (version 1.1.4), pheatmap (version 1.0.12) and viridis (version 0.6.2), as well as foreach (version 1.5.1), doParallel (version 1.0.16), future (version 1.31.0), rprojroot (version 2.0.2), yaml (version 2.2.1) and WriteXLS (version 6.3.0), Analyst 1.7 software, MultiQuant 3.0.3, Metaboanalyst

For manuscripts utilizing custom algorithms or software that are central to the research but not yet described in published literature, software must be made available to editors and reviewers. We strongly encourage code deposition in a community repository (e.g. GitHub). See the Nature Portfolio guidelines for submitting code & software for further information.

Data

Policy information about availability of data

All manuscripts must include a data availability statement. This statement should provide the following information, where applicable:
- Accession codes, unique identifiers, or web links for publicly available datasets
- A description of any restrictions on data availability
- For clinical datasets or third party data, please ensure that the statement adheres to our policy

All requests will be fulfilled by the lead contact Hendrik Poeck (E-mail address: hendrik.poeck@ukr.de). All sequencing data are publicly available at the time of publication. Single cell RNA-seq data of murine organoids were deposited at Gene Expression Omnibus (GEO) (accession number: GSE261714, https://www.ncbi.nlm.nih.gov/geo/query/acc.cgi?acc=GSE261714). The publicly available dataset we utilized for cell type annotation in our single cell RNA-seq data of

murine organoids is available at Gene Expression Omnibus (GEO) (accession number: GSE92332, https://www.ncbi.nlm.nih.gov/geo/query/acc.cgi?acc=GSE92332). Primary mass spectrometry data from patient stool samples have been annotated with clinical metadata and deposited at Zenodo under accession number 6603017 (https://zenodo.org/record/6603017). 16S data were deposited at the European Nucleotide Archive (ENA) (accession number; ERP180829, https://www.ebi.ac.uk/ena/browser/view/ERP180829). The remaining data are available within the Article, Supplementary Information or Source Data file. Any additional information is available from the corresponding authors.

Code availability
The scripts used for scRNA-seq analysis have been deposited at GitHub (https://github.com/lit-regensburg/scRNA_intest_org_metabolites; DOI: 10.5281/zenodo.16847406).

Research involving human participants, their data, or biological material

Policy information about studies with human participants or human data. See also policy information about sex, gender (identity/presentation), and sexual orientation and race, ethnicity and racism.

Reporting on sex and gender	Findings reported in our study apply to both sexes. The study cohort included female and male patients and sex was assigned based on electronic patient records. Detailed numbers of patients in each sex is reported in Supplementary Table 1, male and female patients were equally distributed across both centers.<br>We did not consider a priori sex- or gender-based analysis as they did not affect microbiome-predicted outcomes in published cohorts of allogeneic stem-cell transplantation patients (Taur et al., Blood 2014; Peled et al., NEJM 2020).<br>We included sex as a covariate when comparing patients before (Thiele-Orberg et al., Nature Cancer 2024). However, we did not detect statistically significant differences between groups.<br>Material used for in vitro experiments was derived from both male and female participants (GvHD biopsies) as well as healthy volunteers (PBMCs).
Reporting on race, ethnicity, or other socially relevant groupings	The study cohort included patients enrolled at the University Hospital Rechts der Isar in Munich and the University Hospital in Regensburg. Both centers are located in Bavaria, Germany and treat a populace that is largely of Western and Central European descent. In Munich, epidemiological data regarding place of birth of the patients and their parents was recorded, but race and ethnicity was not recorded a priori. In Regensburg, all patients identified as "Non-hispanic/white" based on self-reporting.
Population characteristics	Patient characteristics including age, sex, diagnosis, donor type, conditioning regimen, stem cell source, incidence of acute GI-GvHD, incidence of TRM, incidence of relapse, and 2-year overall survival are reported in Supplementary Table 1.
Recruitment	At both centers, Munich and Regensburg, patients undergoing allogeneic stem-cell transplantation were enrolled (Munich: 2019-2021, Regensburg 2018-2021) in this prospective, observational cohort after obtaining informed consent and in accordance with IRB-approved study protocols (see "Ethics oversight"). All patients who were admitted for allo-SCT were screened for study enrollment. Stool samples were collected at predetermined time-points (calendar-driven, weekly from Day -7 until Day +35 or discharge, whichever occurred later) or in response to clinical occurrences (event-driven, e.g., onset of acute GI-GvHD). Patient were included in the analysis if a minimum of 3 longitudinal samples were available.<br>We are not aware of a selection bias, since all patients were routinely screened for study participation, and the large majority of patients consented to study participation. There is a potential bias for inclusion of patients with a longer hospital stay in the analysis, since they are more likely to have met the minimum number of samples required.
Ethics oversight	Technical University of Munich, IRB 295/18 S; University of Regensburg, IRB 14-47_1-101, 14-101-0047, 21-2521-01., 21-2224-101

Note that full information on the approval of the study protocol must also be provided in the manuscript.

Field-specific reporting

Please select the one below that is the best fit for your research. If you are not sure, read the appropriate sections before making your selection.

☒ Life sciences ☐ Behavioural & social sciences ☐ Ecological, evolutionary & environmental sciences

For a reference copy of the document with all sections, see nature.com/documents/nr-reporting-summary-flat.pdf

Life sciences study design

All studies must disclose on these points even when the disclosure is negative.

Sample size	The respective sample sizes were determined based on internal pilot studies, other projects, and previous research.
Data exclusions	No data was excluded from the analysis.
Replication	In vitro data were generated in at least three independent experiments. In vivo experiments were repeated at least once.
Randomization	Samples and mice were randomly assigned to groups.

Blinding	Data collection was performed blinded. In vivo metabolite applications were performed unblinded due to the distinctly different appearence of the used metabolites.

Reporting for specific materials, systems and methods

We require information from authors about some types of materials, experimental systems and methods used in many studies. Here, indicate whether each material, system or method listed is relevant to your study. If you are not sure if a list item applies to your research, read the appropriate section before selecting a response.

Materials & experimental systems

n/a	Involved in the study
☐	☒ Antibodies
☐	☒ Eukaryotic cell lines
☒	☐ Palaeontology and archaeology
☐	☒ Animals and other organisms
☐	☒ Clinical data
☒	☐ Dual use research of concern
☒	☐ Plants

Methods

n/a	Involved in the study
☒	☐ ChIP-seq
☐	☒ Flow cytometry
☒	☐ MRI-based neuroimaging

Antibodies

Antibodies used	This information is additionally available in Table S2:<br>m CD25 – PE (AB_395101 ; BD; 1:200) m CD4 – Pacific Blue (AB_2739450 ;BD; 1:200) m CD8a – PerCP/Cyanine5.5 (AB_394081 ; BD; 1:200) m Ly-6C –Pacific Blue (AB_1732090; AB_1732079 ; Biolegend; 1:200) m CD11b –PE/Cyanine7 (AB_394491 ; BD; 1:200) m CD326 – BUV395 (AB_2740020 ; BD;1:200) m CD45 – Alexa Fluor 700 (AB_493714; AB_493715; Biolegend; 1:250)m CD11b – BUV395 (AB_2738276 ; BD; 1:1000) m CD8a – BUV395 (AB_2732919; BD; 1:200) m CD3 – FITC (AB_312660; AB_312661; Biolegend; 1:200) mCD279 (PD-1) – PE (AB_1877232; AB_1877231; Biolegend; 1:250) m CD4 –PerCP/Cyanine5.5 (AB_893330; AB_893324; Biolegend; 1:200) m IFN-γ –PE/Cyanine7 (AB_1595591; AB_2295770; Biolegend; 1:750) m (C57BL) H-2Kb –Brilliant Violet 421 (AB_2876430; Biolegend; 1:200) m (BALB/c) H-2Kd –Brilliant Violet 421 (AB_2565656; Biolegend; 1:200) m/h FoxP3 – AlexaFluor 647 (AB_439749; AB_439750; Biolegend; 1:100) m I-A/I-E – FITC(AB_313320; AB_313321; Biolegend; 1:1500) m CD11c – PE (AB_313776;AB_313777; Biolegend; 1:200) m F4/80 – PE/Cyanine7 (AB_893490;AB_893478; Biolegend; 1:800) m Ly-6C – Brilliant Violet 421 (AB_2562177;AB_2562178; Biolegend; 1:1000) m CD103 – Brilliant Violet 605(AB_2629724; Biolegend; 1:1000) m Ly-6G – Alexa Fluor 647 (AB_1134162;AB_1134159; Biolegend; 1:1500) m CD3 – APC/Cyanine7 (AB_2057374;AB_2242784; Biolegend; 1:200) m CD86 – PE (AB_313150; AB_313151;Biolegend; 1:200) m CD80 – PerCP/Cyanine5.5 (AB_893406; AB_2291392;Biolegend; 1:200) m CD11c – APC/Cyanine7 (AB_830646; AB_830649;Biolegend; 1:300) m CD45 – PerCP/ Cyanine5.5 (AB_893344; AB_893340;Biolegend; 1:500) m CD3 – APC (AB_2561455; AB_2561456; Biolegend; 1:200)m CD3 – Brilliant Violet 421 (AB_10900227; AB_2562553; Biolegend; 1:200)m CD117 – PE (AB_2734235; Biolegend; 1:200) h CD14 – Brilliant Violet421 (AB_2810579; Biolegend; 1:200) h CD209 – APC (AB_1134055; Biolegend;1:200) h CD11c – PerCP (AB_2566656; Biolegend; 1:200) h CD80 – BV605(AB_11123909; Biolegend; 1:200) h CD86 – BV711 (AB_2565834; Biolegend;1:200) h HLA-DR – FITC (Cat. 980402; Biolegend; 1:200) h TNFa – BUV395(AB_2738533; BD; 1:100) h CD25 – BUV737 (AB_2870132; BD; 1:100) h CD8a –BUV805 (AB_2871326; BD; 1:100) h PD-1 – Brilliant Violet 421 (AB_2721517; Biolegend; 1:100) h TIM-3 – Brilliant Violet 605(AB_2741099; BD; 1:100) h T-bet – Brilliant Violet 650 (AB_2738616; BD;1:100) h CD4 – Brilliant Violet 711 (AB_2737965; BD; 1:100) h CD3 –Brilliant Violet 785 (AB_11219196; Biolegend; 1:100) h Perforin – FITC(AB_493252; Biolegend; 1:100) h IFNy – BB700 (AB_2744484; BD; 1:100) hGranzyme B – PE-Dazzle 594 (AB_2728382; Biolegend; 1:100) Foxp3 – PE-Cy5(AB_10597134; Thermo Fisher; 1:50) TotalSeq™-B0301 anti-mouse Hashtag 1– Oligo Hashtag (AB_2814067; Biolegend; 1:100) TotalSeq™-B0302anti-mouse Hashtag 2 – Oligo Hashtag (AB_2814068; Biolegend; 1:100)TotalSeq™-B0303 anti-mouse Hashtag 3 – Oligo Hashtag (AB_2814069;Biolegend; 1:100) TotalSeq™-B0304 anti-mouse Hashtag 4 – Oligo Hashtag(AB_2814070; Biolegend; 1:100) TotalSeq™-B0305 anti-mouse Hashtag 5 –Oligo Hashtag (AB_2814071; Biolegend; 1:100) TotalSeq™-B0306 anti-mouseHashtag 6 – Oligo Hashtag (AB_2814072; Biolegend; 1:100)
Validation	Just commercially available antibodies were used. Validations were perfomed by the supplying company

Eukaryotic cell lines

Policy information about cell lines and Sex and Gender in Research

Cell line source(s)	BA/F3-FLT3-ITD provided by Natalie Köhler. Human or murine primary cells were derived from both male and female individuals.
Authentication	Profiling by highly polymorphic short tandem repeat loci
Mycoplasma contamination	All cell lines tested negative for mycoplasma
Commonly misidentified lines (See ICLAC register)	N/A

Animals and other research organisms

Policy information about studies involving animals; ARRIVE guidelines recommended for reporting animal research, and Sex and Gender in Research

Laboratory animals	C57BL/6J and BALB/c mice were purchased from Janvier-Labs. Mice genetically modified for the interferon-α receptor 1 (B6(Cg)-Ifnar1tm1.2Ees/J; RRID: IMSR_JAX:028288; here referred to as IfnaR−/−), STING (C57BL/6J-Sting1gt/J; RRID:IMSR_JAX:017537; here referred to as STING Goldenticket, STINGGT/GT), MAVS (B6;129-Mavstm1Zjc/J; RRID:IMSR_JAX:008634; here referred to as MAVS-/-), Lgr5-GFP (B6.129P2-Lgr5tm1(cre/ERT2)Cle/J; RRID:IMSR_JAX:008875), STING flox (B6;SJL-Sting1tm1.1Camb/J, RRID:IMSR_JAX:031670) and Vilin-Cre (B6.Cg-Tg(Vil1-cre)997Gum/J, RRID:IMSR_JAX:004586) have been described previously.
Wild animals	No wild animals involved
Reporting on sex	In vivo experiments were performed with female mice. Organs for in vitro experiments were derived from female and male animals.
Field-collected samples	No field-collected samples involved
Ethics oversight	The study was approved by the ethik committee of Regierung von Oberbayer and Regierung von Unterfranken.

Note that full information on the approval of the study protocol must also be provided in the manuscript.

Clinical data

Policy information about clinical studies

All manuscripts should comply with the ICMJE guidelines for publication of clinical research and a completed CONSORT checklist must be included with all submissions.

Clinical trial registration	The observational cohort reported in this study is registered under the German Clinical Trials Register DRKS00034175.
Study protocol	There is no study protocol available. Biosamples and data were collected as described below.
Data collection	We prospectively collected stool samples at calendar-driven and event-driven time-points for characterization by mass-spectrometry for expression of microbiota-derived metabolites DAT and ICA. The entirety of data collection has been described before (Thiele-Orberg et al., 2024).
Outcomes	As primary outcome, overall survival (OS) and cumulative incidences (adjusted for competing risks) of transplant-related mortality (TRM), relapse and GvHD were prospectively recorded until death or event occurrence.

Plants

Seed stocks	N/A
Novel plant genotypes	N/A
Authentication	N/A

Flow Cytometry

Plots

Confirm that:

- [x] The axis labels state the marker and fluorochrome used (e.g. CD4-FITC).
- [x] The axis scales are clearly visible. Include numbers along axes only for bottom left plot of group (a 'group' is an analysis of identical markers).
- [x] All plots are contour plots with outliers or pseudocolor plots.
- [x] A numerical value for number of cells or percentage (with statistics) is provided.

Methodology

Sample preparation	different methods were used depending on (In vitro or in vivo) samples. Methods are detailed specified in the Material and Methods section.
Instrument	FACS LSR Fortessa X-20 or Symphony A5 (BD Biosciences)

Software | FACS Diva for collection of data, FlowJo 10.8.1 for analysis

Cell population abundance | *Describe the abundance of the relevant cell populations within post-sort fractions, providing details on the purity of the samples and how it was determined.*

Gating strategy | Gating strategies for all flow cytometry experiments are specified in the supplemental information

☒ Tick this box to confirm that a figure exemplifying the gating strategy is provided in the Supplementary Information.
